# Reversible Cerebral Vasculopathy Resembling Reversible Cerebral Vasoconstriction Syndrome in a Case of Untreated Basedow’s (Graves') Disease

**DOI:** 10.7759/cureus.98600

**Published:** 2025-12-06

**Authors:** Miwako Ishikawa, Tomoaki Kameda, Akiko Deguchi, Kazuhiro Saito, Shigeru Fujimoto

**Affiliations:** 1 Division of Neurology, Department of Medicine, Jichi Medical University, Shimotsuke, JPN; 2 Department of Neurology, Shin-Oyama City Hospital, Oyama, JPN; 3 Department of Diabetes and Endocrinology, Shin-Oyama City Hospital, Oyama, JPN

**Keywords:** basedow’s disease, moyamoya vasculopathy, reversible cerebral vasoconstriction syndrome, thunderclap headache, thyrotoxicosis

## Abstract

Reversible cerebral vasoconstriction syndrome (RCVS) involves transient, multifocal cerebral artery narrowing. We report the case of a 42-year-old woman presenting with thunderclap headaches one month after thyrotoxicosis onset. Brain computed tomography and magnetic resonance imaging (MRI) were unremarkable, but magnetic resonance angiography (MRA) revealed focal narrowing of the M2 segment of the right middle cerebral artery. Initial treatment with intravenous nicardipine failed to relieve symptoms. After diagnosing Basedow’s (Graves') disease and initiating antithyroid therapy, the headaches markedly improved. This case suggests that RCVS may be triggered by thyrotoxicosis alone and that RCVS secondary to Graves' disease may resolve with appropriate antithyroid treatment.

## Introduction

Basedow’s (Graves') disease involves reversible cerebral vasoconstriction due to thyrotoxicosis and autoimmunity [1]; this is also termed Graves' disease-related moyamoya syndrome. Distinct from idiopathic moyamoya disease, this syndrome often improves with antithyroid or immunosuppressive therapy [1-4]. Reversible cerebral vasoconstriction syndrome (RCVS) is another pathology that shows reversible cerebral vasoconstriction and presents with thunderclap headache. No previous reports of Graves’ disease show RCVS meeting the diagnostic criteria for induced Graves’ disease alone. We provide here the first report of RCVS in an untreated woman in her 40s with Graves’ disease.

## Case presentation

A 42-year-old woman who had been undergoing investigation of hyperthyroidism for a month was transferred to the emergency department with thunderclap headaches and nausea. She was receiving atenolol at 50 mg s.i.d. Her blood pressure was 169/84 mmHg, heart rate was 112 beats/min, and body temperature was 37.7 °C. The initial manifestations of thyrotoxicosis included palpitations, bilateral lower-leg edema, and hypertension. She had no prior history of hypertension. She was alert and oriented, with no focal neurological deficits or meningeal signs evident on examination.

Findings from the investigations laboratory were unremarkable, except for thyroid function tests (Table 1). The mild elevation of hepatic transaminases in this case was suspected to be attributable to thyrotoxicosis.

**Table 1:** Results of laboratory tests on admission to our hospital PT-INR: prothrombin time-international normalized ratio; TSH: thyroid-stimulating hormone, Tg-Ab: anti-thyroglobulin antibody, TPO-Ab: anti-thyroid peroxidase antibody, TRAb: thyroid-stimulating hormone receptor antibody

Laboratory tests	Results	Normal range
White blood cell counts (/μL)	6260	4000-9500
Hemoglobin (g/dL)	11.9	12.0-15.5
Platelet count (×10^3^/μL)	243	130-400
Aspartate aminotransferase (U/L)	64	8-38
Alanine aminotransferase (U/L)	94	4.0-30
Blood urea nitrogen (mg/dL)	8.7	8.0-20.0
Creatinine (mg/dL)	0.35	0.4-0.8
C-reactive protein (mg/dL)	0.1	0.0-0.3
Activated partial thromboplastin time (sec)	24.6	23.0-35.0
PT-INR	1.19	0.90-1.10
D-dimer (μg/mL)	2.5	0-1.0
TSH (μIU/mL)	0	0.35-4.94
Free T3 (pg/mL)	>20	1.68-3.67
Free T4 (ng/mL)	>5	0.7-1.48
Tg-Ab (ng/mL)	8.3	0-4.11
TPO-Ab (IU/mL)	38.3	0-5.61
TRAb (IU/L)	18.5	0-1.9

**Figure 1:**
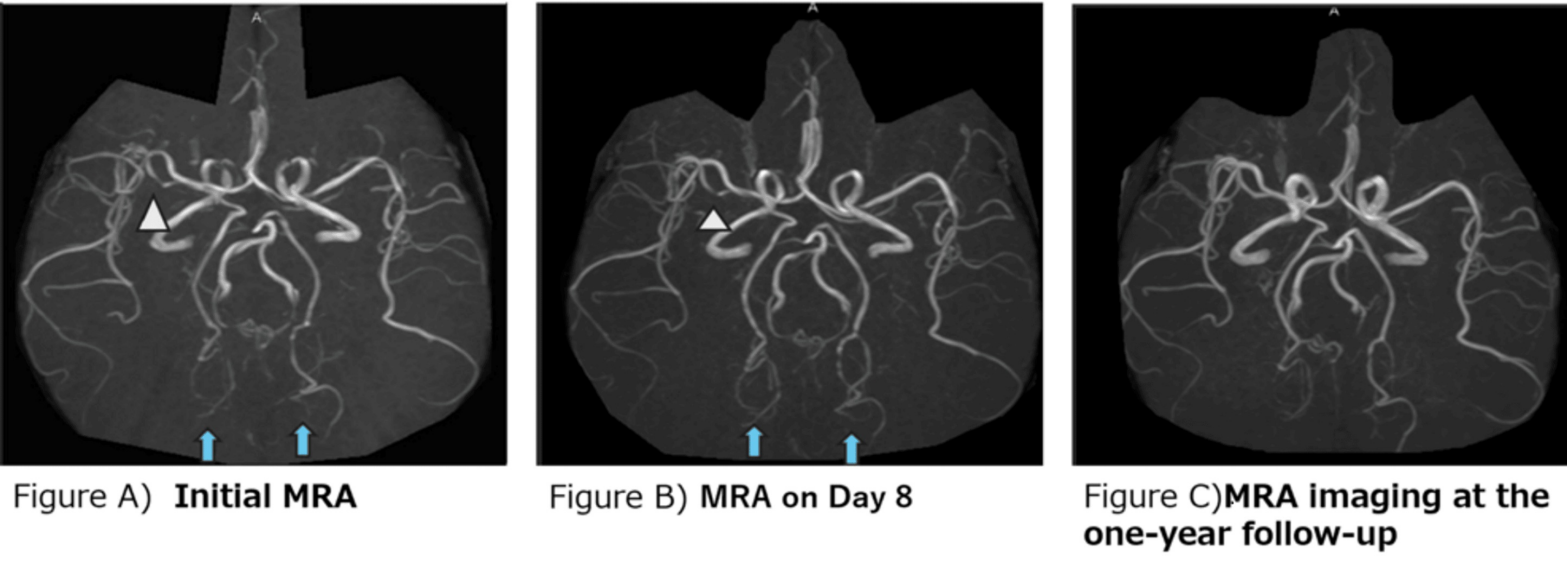
Figures A-C Figure A) MRA shows focal narrowing of the M2 segment of the right middle cerebral artery (arrowhead). Irregularities of bilateral distal posterior cerebral arteries are also visible (arrows). Figure B) Focal narrowing of the M2 segment is still evident (arrowhead). Irregularities of bilateral distal posterior cerebral arteries have improved slightly (arrows). Figure C) The right middle cerebral artery and bilateral posterior cerebral arteries appear normal. MRA: magnetic imaging angiography

Non-contrast-enhanced computed tomography (CT) of the brain showed no abnormalities. Likewise, magnetic resonance imaging (MRI) of the brain showed normal results, with no vasogenic or cytotoxic edema on T2-weighted fluid-attenuated inversion recovery. Magnetic resonance angiography (MRA) showed focal narrowing of the M2 segment of the right middle cerebral artery (Figure 1, A).

Irregularities were also observed in the distal portions of bilateral posterior cerebral arteries. Imaging findings supported a diagnosis of RCVS. No evidence of moyamoya vessels was observed on MRA. Lumbar puncture was not performed in this case. According to the referral document, color flow Doppler sonography had shown diffuse enlargement of the thyroid and diffuse hypervascularity. From these results, the patient was diagnosed with Graves' disease. MRA on hospital day 11 clearly showed multiple foci of narrowing and dilatation in the P2 segments of bilateral posterior cerebral arteries (Figure 1B).​​​

**Figure 2:**
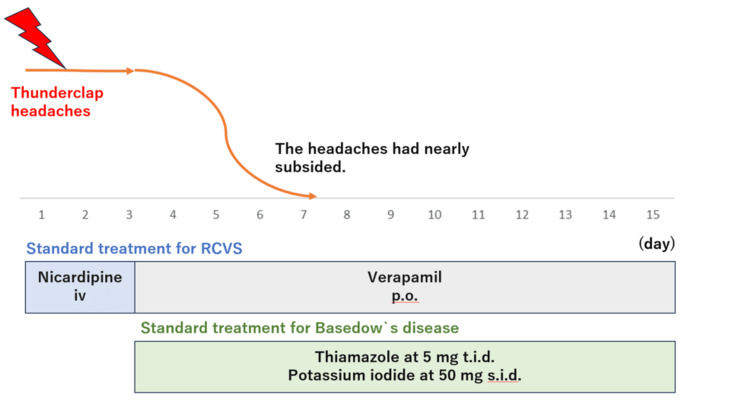
Overview of the clinical course The clinical course of the patient. The thunderclap headache improved after the initiation of antithyroid therapy.

Other diagnoses that had to be considered included subarachnoid hemorrhage (SAH) and primary or secondary central nervous system (CNS) vasculitis [1]. SAH was considered unlikely due to the normal results from non-contrast-enhanced CT and MRI of the brain [5]. Similarly, although primary CNS vasculitis is one of the most important differential diagnoses for RCVS, the probability of primary angiitis of the CNS is considered extremely low if MRI of the brain shows no abnormalities [6]. The observed clinical features met the diagnostic criteria for headache attributable to RCVS. An RCVS score >=5 is considered to offer 99% specificity and 90% sensitivity for diagnosing RCVS, and the score was 6 in this case [7]. One limitation of the diagnosis was the lack of cerebrospinal fluid findings. The lumbar puncture could not be performed because the patient declined the procedure. However, laboratory findings, such as white blood cells, neutrophils, and C-reactive protein, were within normal limits. The possibility of meningitis was considered very low. Continuous infusion of nicardipine hydrochloride was started based on the diagnosis of RCVS, but symptoms remained unimproved. Based on the diagnosis of Graves' disease, oral administration of thiamazole at 5 mg t.i.d. and potassium iodide at 50 mg s.i.d. was started on hospital day 3. At the same time, nicardipine hydrochloride was switched to verapamil hydrochloride. After starting this regimen, symptoms gradually resolved over the course of a few days. The patient was discharged after two weeks of hospitalization (Figure 2).

Follow-up MRA after one year showed normal appearances of the right middle cerebral artery and bilateral posterior cerebral arteries (Figure 1C).

The patient has experienced no further focal neurological deficits or recurrence of RCVS in two years.

## Discussion

This case provides important clinical insights into the association between untreated Graves’ disease and RCVS. First, thyrotoxicosis caused by Graves' disease can trigger cerebral vasoconstriction, which may improve with antithyroid agents. Second, RCVS may be induced solely by thyrotoxicosis in the absence of other known precipitants. Finally, prompt management of thyrotoxicosis may help prevent neurological complications such as RCVS and ischemic stroke.

Previous reports have described associations between Graves’ disease and intracranial arterial stenosis or occlusion, typically detected during evaluation of transient ischemic attacks or stroke [4]. Although headache was the most common symptom in moyamoya syndrome induced due to Graves' disease [5], no reports have described the presence of acute thunderclap headache. Radiologically, these cases often exhibit bilateral or unilateral narrowing of intracranial vessels with acute ischemic lesions. Moyamoya syndrome secondary to Graves’ disease has also been reported. No reports were found describing imaging differences between Graves' disease-associated moyamoya syndrome and RCVS. However, in the present case, the vasoconstriction of the bilateral posterior cerebral arteries progressed centripetally, which is a characteristic finding of RCVS [6].

Moyamoya syndrome associated with Graves’ disease represents a distinct clinical entity that differs from idiopathic moyamoya disease. Unlike idiopathic moyamoya disease [7], Graves' disease-related vasculopathy often improves with normalization of thyroid function or immunotherapy [8-11]. Neurological symptoms and re-narrowing of vessels upon deterioration of thyroid function have also been observed [2,12]. It is possible that the pathophysiological mechanisms of Graves' disease-associated moyamoya disease and RCVS may share certain similarities.

The pathogenesis of vasoconstriction in Graves' disease likely involves thyrotoxicosis-induced autonomic imbalance [8]. While several reports have described RCVS in the context of postpartum thyrotoxicosis [13] or drug exposure (e.g., teprotumumab [14]), no prior reports have documented thunderclap headaches or angiographic reversibility solely due to untreated Graves' disease. A PubMed search revealed no previous reports of reversible cerebral vasoconstriction syndrome (RCVS) induced solely by thyrotoxicosis due to Graves' disease. Notably, in our patient, thunderclap headaches resolved before achieving euthyroidism, suggesting that removal of the precipitating factor (i.e., thyrotoxicosis) was therapeutic.

RCVS is characterized by the sudden onset of thunderclap headaches and reversible segmental cerebral vasoconstriction [4]. The proposed mechanisms include sympathetic overactivity, endothelial dysfunction, oxidative stress, and genetic predisposition [15]. These factors overlap with the pathophysiological changes observed in Graves' disease, including altered sympathovagal balance and vascular endothelial damage [16]. Studies of heart rate variability have shown consistent patterns of autonomic imbalance in both RCVS and Graves' disease [17,18]. While β-blockers may not normalize the autonomic dysfunction in Graves' disease, antithyroid treatment appears to restore autonomic balance, potentially reducing the risk of RCVS [18].

In the present case, treatment initiation was delayed due to plans for thyroid scintigraphy to rule out Plummer’s disease, despite typical findings of Graves' disease (e.g., diffuse goiter, elevated thyrotropin receptor antibodies, and increased vascularity on ultrasound). This delay likely prolonged the exposure to thyrotoxicosis. Fatal strokes have been reported in association with Graves’ disease-related vasculopathy [4,19-20], underscoring the need for timely management.

## Conclusions

In conclusion, thyrotoxicosis caused by untreated Basedow’s (Graves') disease can cause RCVS as well as moyamoya syndrome. Early initiation of antithyroid therapy may alleviate symptoms and prevent cerebrovascular complications. Clinicians should thus remain vigilant for neurological symptoms in hyperthyroid patients and initiate treatment without unnecessary delay.
